# Assessment of Public Awareness of Cellulitis in Al-Qunfudhah Region, Saudi Arabia

**DOI:** 10.7759/cureus.63163

**Published:** 2024-06-25

**Authors:** Medhat Taha, Mazen Mohammed Minaji Alzelaei, Ali Mohammed Salem Al-Qarni, Mohammed Ahmed Muhanni Al-Ammari, Hassan Shulaymi Thakir Algamdi, Abdullah Amer Ibrahim Almaeidi, Hassan Abdu Ali Al-Faqih

**Affiliations:** 1 Department of Anatomy, Umm Al-Qura University, Al-Qunfudhah, SAU; 2 College of Medicine and Surgery, Umm Al-Qura University, Al-Qunfudhah, SAU

**Keywords:** knowledge, saudi arabia, population, attitude, awareness, prevalence, cellulitis

## Abstract

Background: The term "cellulitis" is frequently used to describe a non-necrotizing inflammation of the skin and subcutaneous tissues that is typically caused by an acute infection and does not affect the muscles or fascia. Warmth, erythema, tenderness, swelling, and localized pain are the hallmarks of cellulitis. Life-threatening and debilitating outcomes from cellulitis include necrotizing fasciitis, necrotizing hypodermitis, abscess formation, septic shock, and, in extreme cases, death. The current study aimed to assess public awareness of cellulitis in the Al-Qunfudhah region, Saudi Arabia.

Methods: A descriptive cross-sectional study was conducted targeting all residents in Al-Qunfudhah, Saudi Arabia, during the period from January to March 2024. Data were collected using a pre-structured online questionnaire. The study questionnaire included participants' demographic data, cellulitis data, and knowledge and attitude towards cellulitis. The final questionnaire was uploaded online using social media platforms by the researchers and their friends until no more new participants were included.

Results: A total of 470 records were analyzed. Among the respondents, the majority were male (n=347, 73.8%), and the highest proportion fell within the age range of 18 to 25 years (n=174, 37.0%). The highest proportion of respondents correctly identified cellulitis as a medical condition affecting the skin and soft tissues underneath it, typically caused by infection or injury (n=278, 59.1%). Additionally, the majority acknowledged that cellulitis can cause pain, swelling, and redness in the affected area (n=240, 51.1%). As for factors associated with awareness of cellulitis, significant associations were found with age group (p=0.031), educational levels (p=0.003), and employment status (p=0.002).

Conclusions: This study revealed a high level of awareness of cellulitis among participants, especially highly educated and employed participants. Participants believed that healthcare providers play a crucial role in raising population awareness of cellulitis health problems.

## Introduction

Cellulitis is one of the most typical infections of the skin and soft tissues [1-3]. As a result of bacterial invasion through breaches in the skin barrier, cellulitis, which includes erysipelas, presents as an area of skin erythema, edema, and warmth. [4] Cellulitis is frequently misdiagnosed [5]; hence, other diagnoses ought to be considered. As a common skin infection, cellulitis affects both the epidermis and subcutaneous tissue, resulting in pain, edema, redness, and warmth in the affected area [6]. As many as 70-80% of cases involve the lower limbs, although it can occur in other body regions [7]. Gram-positive bacteria, such as *Staphylococcus aureus* and beta-hemolytic streptococcus, are responsible for most occurrences of cellulitis [6,8,9]. Cellulitis is a serious medical emergency that frequently necessitates hospitalization, can cause recurring illnesses, and can have long-term morbidity [10].

Cellulitis is characterized by erythema (redness), warmth to the touch, edema (swelling), and tenderness or discomfort in specific areas [11]. There may be a fever, chills, and malaise, along with lymphangitis and/or bacteremia [1,3]. Infection of burns, wounds, surgical incisions, or skin lesions is typically the cause of cellulitis [11,12]. Cellulitis can affect any part of the body in adults, but it typically affects the skin on the arms and lower legs. Children's cellulitis usually affects the lower limbs, periorbital infection, and extremities [13]. Numerous bacteria, most frequently *Streptococcus pyogenes* (*Strep A*), *Staphylococcus aureus*, or other beta-hemolytic streptococci, can cause cellulitis. Recurrent episodes of cellulitis affect about one-third of patients [14,15]. Low-dose antibiotics taken over an extended period of time have been demonstrated to lower the chance of recurrence, but successful preventative measures still need to be found [15].

Cellulitis can cause life-threatening and incapacitating consequences, such as necrotizing fasciitis, necrotizing hypodermitis, abscess development, septic shock, and, in severe cases, death, if diagnosis and treatment are delayed [16-18]. Determining the risk factors linked to cellulitis is essential for taking preventative action. Venous edema, lymphedema, skin disorders, traumatic injuries, leg ulcers, peripheral vascular disease, fungal infections, a history of cellulitis, and obesity are examples of risk factors [19,20]. Our research work aimed to measure awareness of the Al-Qunfudhah population of cellulitis health problems.

## Materials and methods

Methodology

A descriptive cross-sectional study was conducted targeting all residents in Al-Qunfudhah, Saudi Arabia, during the period from January to March 2024. Adults aged above 18 years in Al-Qunfudhah Governorate were included, while children <18 years and who live outside Al-Qunfudhah Governorate and those who refused to participate or had missing questionnaire data, as well as participants who might have a conflict of interest, were excluded. Our study sample size of 501 was calculated with the aid of the Raosoft calculator (Raosoft, Inc., Seattle, WA) with a 5% margin of error and 95% confidence level (CI). Data were collected using a pre-structured online questionnaire that was initiated by the study authors with the help of experts and a literature review. A panel of three experts in the study field reviewed the initial questionnaire after its running in a pilot study to assess its content validity and applicability, and all suggested changes were applied. The final questionnaire included participants' demographic data, history of diagnosis with cellulitis, and associated complaints. The third section covered their awareness of cellulitis, including disease definition, causes, clinical symptoms, diagnosis methods, management methods, and associated complications, besides their source of information. The last section covered participants’ attitudes towards cellulitis and the role of health care staff. The final questionnaire was uploaded online using social media platforms by the researchers and their friends until no more new participants were included. Ethical approval was obtained from the Biomedical Research Ethics Committee of Umm Al-Qura University, Al-Qunfudhah, Saudi Arabia (approval number: HAPO-02-K-012-2024-02-1980).

Statistical analysis

The statistical analysis was conducted using RStudio software version 4.3.1 (Posit Software, PBC, Boston, MA, USA). Descriptive statistics were employed to summarize variables, including frequency distributions and percentages. Inferential statistics, including Pearson's chi-squared test and Fisher's exact test, were utilized to assess the associations between demographic variables and being aware of and diagnosed with cellulitis. Additionally, binary logistic regression analysis was performed to identify the independent predictors of being aware of and diagnosed with cellulitis in separate models. The models included variables that showed significant associations in the inferential analysis. Odds ratios (ORs) with 95% confidence intervals (CIs) were computed to quantify the strength and direction of associations. A significance level of p<0.05 was considered statistically significant.

## Results

Sociodemographic characteristics

Initially, we received the responses of 501 participants. However, 31 records of participants aged <18 years were excluded. Therefore, a total of 470 records were analyzed. Among the respondents, the majority were male (n=347, 73.8%), and the highest proportion fell within the age range of 18 to 25 years (n=174, 37.0%). Regarding educational levels, the largest group consisted of individuals with a university education (n=321, 68.3%). In terms of employment status, a slight majority were employed (n=251, 53.4%). Additionally, almost all participants were Saudi nationals (n=469, 99.8%, Table 1).

**Table 1:** Sociodemographic characteristics

Characteristic	N (%)
Gender	
Male	347 (73.8%)
Female	123 (26.2%)
Age (years)	
18 to 25	174 (37.0%)
26 to 35	145 (30.9%)
36 to 45	105 (22.3%)
46 to 55	35 (7.4%)
>55	11 (2.3%)
Educational levels	
Illiterate	1 (0.2%)
Primary	10 (2.1%)
Middle school	11 (2.3%)
Secondary	90 (19.1%)
University	321 (68.3%)
Diploma	4 (0.9%)
Master's	26 (5.5%)
PhD	7 (1.5%)
Employment status	
Non-employed	219 (46.6%)
Employed	251 (53.4%)
Nationality	
Saudi	469 (99.8%)
Non-Saudi	1 (0.2%)

Participants’ awareness of cellulitis

Less than half of the participants had ever heard about cellulitis (n=231, 49.1%). The highest proportion of respondents correctly identified cellulitis as a medical condition affecting the skin and soft tissues underneath it, typically caused by infection or injury (n=278, 59.1%). Additionally, the majority acknowledged that cellulitis can cause pain, swelling, and redness in the affected area (n=240, 51.1%). Among the participants, the most commonly recognized method for diagnosing cellulitis was all the available options (n=225, 47.9%), including visual examination of the skin and surrounding tissues for signs of swelling and redness, blood tests to detect inflammation and infection, imaging techniques such as X-rays or CT scans for internal tissue evaluation, and the collection of fluid samples from affected areas for infection analysis and identification. Regarding treatments, the highest proportion of respondents identified all the available treatments (n=240, 51.1%), including treatment with antibiotics to combat potential bacterial infections, the utilization of anti-inflammatory medications to alleviate pain and swelling, advocating rest and sustained elevation of the affected limb, and considering surgical intervention in instances of severe skin breakdown.

**Figure 1:**
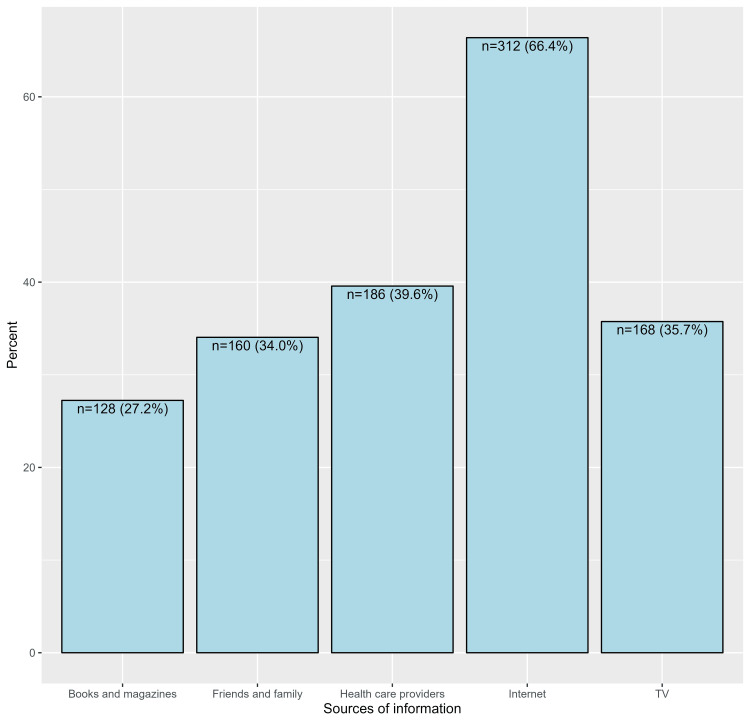
Proportions of sources of information regarding cellulitis

Moreover, the majority of participants correctly indicated that cellulitis can appear anywhere on the skin (n=384, 81.7%) and that it is commonly caused by a bacterial skin infection affecting deeper layers of the skin and underlying tissues (n=399, 84.9%). Additionally, a notable proportion of respondents correctly recognized that cellulitis is not a contagious disease (n=203, 43.2%). Furthermore, most participants recognized that if left untreated, cellulitis can spread to other parts of the body (n=390, 83.0%) and can quickly become a life-threatening condition (n=368, 78.3%, Table 2). In general, the most common sources of information regarding cellulitis were the Internet (n=312, 66.4%), followed by healthcare providers (n=186, 39.6%) and television (n=168, 35.7%, Figure 1).

**Table 2:** Participants’ awareness of cellulitis * indicates correct answers

Characteristic	N (%)
Ever heard about cellulitis	231 (49.1%)
What is cellulitis?	
Cellulitis is a medical condition that affects the skin and the soft tissues underneath it, usually caused by infection or injury*	278 (59.1%)
Cellulitis is a disorder that affects the nervous tissue in the body and can lead to loss of sensation or movement	112 (23.8%)
Cellulitis is a condition that affects blood vessels in the body, causing swelling and pain	66 (14.0%)
Cellulitis is a digestive problem that affects the stomach and intestines, causing inflammation and ulcers	14 (3.0%)
Effects of cellulitis on health	
Cellulitis can cause localized increases in temperature and redness	144 (30.6%)
Cellulitis can cause pain, swelling, and redness in the affected area*	240 (51.1%)
Cellulitis can lead to loss of movement or the ability to use the affected limb	60 (12.8%)
Cellulitis can cause digestive disturbances and changes in sleep patterns	26 (5.5%)
How can a doctor diagnose cellulitis?	
Visual examination of the skin and surrounding tissues for signs of swelling and redness	91 (19.4%)
Blood tests to check for signs of inflammation and infection	77 (16.4%)
Imaging tests such as X-rays or CT scans to evaluate internal tissues	50 (10.6%)
Taking a sample of fluid from the affected area for analysis and identification of the infection	27 (5.7%)
A doctor may use all of the above options*	225 (47.9%)
What are the available treatments for cellulitis?	
Treatment with antibiotics to address potential bacterial infection	108 (23.0%)
Use of anti-inflammatory medications to reduce pain and swelling	68 (14.5%)
Rest and continuous elevation of the affected limb	32 (6.8%)
In cases of severe skin breakdown, surgery may be necessary	22 (4.7%)
All of the above options are possible*	240 (51.1%)
Can cellulitis appear anywhere on the skin?	384 (81.7%)
If left untreated, can the infection spread to other parts of the body?	390 (83.0%)
If cellulitis is left untreated, can it quickly become a life-threatening condition?	368 (78.3%)
Do you think cellulitis is a contagious disease?	203 (43.2%)
Is cellulitis commonly caused by a common bacterial skin infection affecting the deeper layers of the skin and underlying tissues?	399 (84.9%)

Factors associated with awareness of cellulitis

In the analysis of factors associated with awareness of cellulitis, significant associations were found with age group (p=0.031), educational levels (p=0.003), and employment status (p=0.002). Notably, individuals aged 26 to 35 years displayed a lower proportion of awareness (n=61, 42.1%) compared to those aged 18 to 25 years. Similarly, the employed group exhibited a higher proportion of awareness (n=140, 55.8%) compared to the non-employed group. Regarding educational levels, individuals with a PhD demonstrated a significantly higher proportion of awareness (n=6, 85.7%) compared to those with an illiterate/primary education. In the multivariable analysis, individuals aged 26 to 35 years had lower odds of being aware of cellulitis compared to those aged 18 to 25 years (OR=0.49, 95% CI: 0.29 to 0.81, p=0.006). Furthermore, employed individuals had higher odds of awareness compared to non-employed individuals (OR=1.90, 95% CI: 1.21 to 3.01, p=0.006, Table 3).

**Table 3:** Factors associated with being aware of cellulitis Pearson's chi-squared test; Fisher's exact test OR: odds ratio, CI: confidence interval

Characteristic	Aware about cellulitis			Multivariable regression		
	No N=239	Yes N=231	p-value	OR	95% CI	p-value
Gender			0.118			
Male	169 (48.7%)	178 (51.3%)				
Female	70 (56.9%)	53 (43.1%)				
Age (years)			0.031			
18 to 25	88 (50.6%)	86 (49.4%)		Reference	Reference	
26 to 35	84 (57.9%)	61 (42.1%)		0.49	0.29, 0.81	0.006
36 to 45	53 (50.5%)	52 (49.5%)		0.58	0.32, 1.04	0.07
46 to 55	11 (31.4%)	24 (68.6%)		1.23	0.51, 3.07	0.65
> 55	3 (27.3%)	8 (72.7%)		3.22	0.71, 19.9	0.157
Educational levels			0.003			
Illiterate/primary	7 (63.6%)	4 (36.4%)		Reference	Reference	
Middle school	4 (36.4%)	7 (63.6%)		3.29	0.49, 27.1	0.236
Secondary	61 (67.8%)	29 (32.2%)		1.22	0.28, 6.90	0.8
University	152 (47.4%)	169 (52.6%)		2.85	0.69, 15.7	0.176
Diploma	3 (75.0%)	1 (25.0%)		0.88	0.03, 12.2	0.929
Master's	11 (42.3%)	15 (57.7%)		2.96	0.58, 19.2	0.213
PhD	1 (14.3%)	6 (85.7%)		12.2	1.17, 315	0.061
Employment status			0.002			
Non-employed	128 (58.4%)	91 (41.6%)		Reference	Reference	
Employed	111 (44.2%)	140 (55.8%)		1.9	1.21, 3.01	0.006
Nationality			0.491			
Saudi	239 (51.0%)	230 (49.0%)				
Non-Saudi	0 (0.0%)	1 (100.0%)				

Participants’ knowledge of different aspects of cellulitis

The highest level of awareness of symptoms of cellulitis was about recognizing pain in the affected area (n=386, 82.1%) and redness and warmth of the affected skin (n=367, 78.1%). Additionally, participants demonstrated strong knowledge of factors that reduce the risk of cellulitis, including cleaning and caring for wounds (n=416, 88.9%) and paying attention to signs of infection in wounds (n=417, 89.1%). Notably, a substantial proportion of respondents demonstrated awareness of potential complications, with 66.4% (n=312) recognizing bloodstream infection, 60.4% (n=284) acknowledging gangrene (tissue death), and 41.7% (n=196) identifying bone infection. Regarding risk factors for developing cellulitis, participants displayed considerable awareness, with injury or cut being the most recognized risk factor (n=322, 68.5%), followed by a weakened immune system (n=280, 59.6%) and skin conditions (n=264, 56.2%, Table 4).

**Table 4:** Participants’ knowledge of different aspects of cellulitis * indicates a multiple-choice item

Characteristic	N (%)
Symptoms of cellulitis	
Pain in the affected area	386 (82.1%)
Redness and warmth of the affected skin	367 (78.1%)
Swelling of the skin	358 (76.2%)
Fever and chills	339 (72.1%)
Risk factors for developing cellulitis*	
Arterial diseases	150 (31.9%)
Injury or cut	322 (68.5%)
Obesity	154 (32.8%)
Previous history of cellulitis	242 (51.5%)
Skin conditions	264 (56.2%)
Weakened immune system	280 (59.6%)
Complication of cellulitis*	
Bloodstream infection	312 (66.4%)
Bone infection	196 (41.7%)
Endocarditis	148 (31.5%)
Gangrene (tissue death)	284 (60.4%)
Joint infection	192 (40.9%)
Factors that reduce the risk of cellulitis	
Moisturizing the skin	384 (82.9%)
Wearing properly fitting closed-toe shoes	342 (73.4%)
Trimming nails carefully and correctly	353 (75.9%)
Cleaning and caring for wounds	416 (88.9%)
Paying attention to signs of infection in wounds	417 (89.1%)

Prevalence of cellulitis and the associated factors

**Figure 2:**
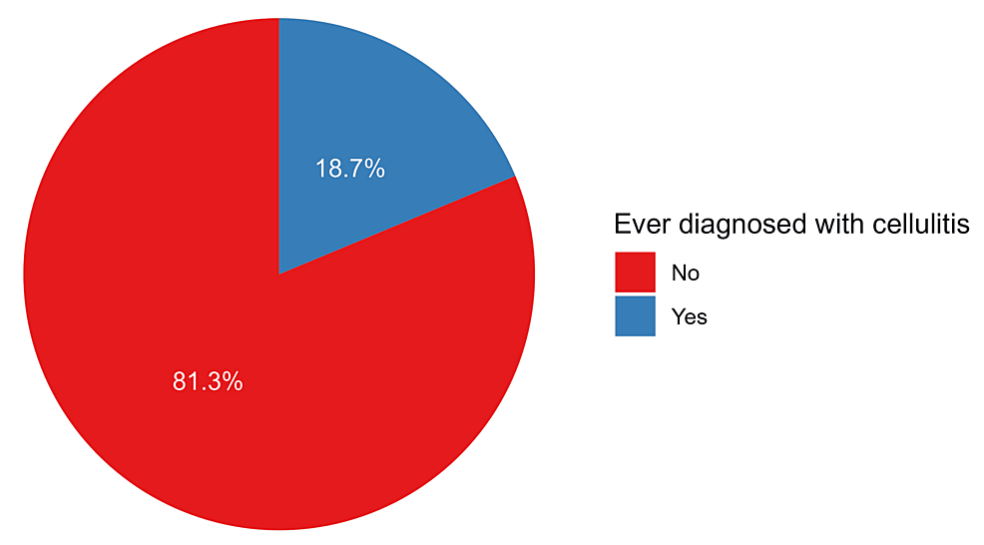
Proportions of participants who had ever been diagnosed with cellulitis

Among the participants, 88 respondents had ever been diagnosed with cellulitis (18.7%, Figure 2). Of those who were diagnosed with cellulitis, the most common sites for cellulitis were the lower limbs (n=46, 52.4%) and the upper limbs (n=40, 45.2%), whereas only 2.4% had cellulitis around the eyes. Notably, a higher proportion of males (n=74, 21.3%) were diagnosed with cellulitis compared to females (n=14, 11.4%, p=0.015). Additionally, employed individuals exhibited a significantly higher proportion of cellulitis diagnoses (n=63, 25.1%) compared to non-employed individuals (n = 25, 11.4%). Additionally, the diagnosis of cellulitis increased significantly with advanced age, from 15.5% (n=27) among participants aged 18 to 25 years and 14.5% (n=21) for those aged 26 to 35 to 26.7% (n=28), 20.0% (n=7), and 45.5% (n=5) among 36 to 45 years, 46 to 55, and >55 years, respectively (p=0.017). Importantly, on the multivariable analysis, only employment status was a significant predictor of cellulitis diagnosis, where employed participants were more likely to experience cellulitis compared to their non-employed counterparts (OR=2.54, 95% CI: 1.38 to 4.81, p=0.003, Table 5).

**Table 5:** Factors associated with being diagnosed with cellulitis Pearson's chi-squared test; Fisher's exact test OR: odds ratio, CI: confidence interval

	Ever diagnosed with cellulitis			Multivariable regression		
Characteristic	No N=382	Yes N=88	p-value	OR	95% CI	p-value
Gender			0.015			
Male	273 (78.7%)	74 (21.3%)		Reference	Reference	
Female	109 (88.6%)	14 (11.4%)		0.73	0.37, 1.40	0.363
Age (years)			0.017			
18 to 25	147 (84.5%)	27 (15.5%)		Reference	Reference	
26 to 35	124 (85.5%)	21 (14.5%)		0.60	0.30, 1.18	0.139
36 to 45	77 (73.3%)	28 (26.7%)		1.11	0.56, 2.19	0.772
46 to 55	28 (80.0%)	7 (20.0%)		0.79	0.28, 2.03	0.632
>55	6 (54.5%)	5 (45.5%)		3.38	0.87, 12.5	0.068
Educational levels			0.161			
Illiterate/ primary	7 (63.6%)	4 (36.4%)				
Middle school	9 (81.8%)	2 (18.2%)				
Secondary	74 (82.2%)	16 (17.8%)				
University	266 (82.9%)	55 (17.1%)				
Diploma	4 (100.0%)	0 (0.0%)				
Master's	18 (69.2%)	8 (30.8%)				
PhD	4 (57.1%)	3 (42.9%)				
Employment status			<0.001			
Non-employed	194 (88.6%)	25 (11.4%)		Reference	Reference	
Employed	188 (74.9%)	63 (25.1%)		2.54	1.38, 4.81	0.003
Nationality			0.187			
Saudi	382 (81.4%)	87 (18.6%)				
Non-Saudi	0 (0.0%)	1 (100.0%)				

Attitudes towards cellulitis

The internal consistency of items related to participants’ attitudes (n=4) was 0.788 as per the outcomes of the Cronbach’s alpha test, indicating good reliability. In Table 6, participants' attitudes towards cellulitis are outlined. The majority strongly agreed that healthcare provides sufficient attention to raising awareness of cellulitis (n=323, 68.7%) and that general awareness of cellulitis can contribute to better prevention (n=339, 72.1%). Additionally, a significant proportion strongly agreed that it is necessary to visit a doctor when noticing rapid increases in skin redness, accompanied by elevated temperature, chills, swelling, and pain in the skin (n=351, 74.7%). Moreover, the majority strongly agreed that more education and awareness of cellulitis should be provided (n=347, 73.8%, Table 6).

**Table 6:** Attitudes towards cellulitis

Characteristic	N (%)
Healthcare provides sufficient attention to raising awareness of cellulitis	
Strongly disagree	7 (1.5%)
Disagree	16 (3.4%)
Neutral	31 (6.6%)
Agree	93 (19.8%)
Strongly agree	323 (68.7%)
General awareness of cellulitis can contribute to better prevention	
Strongly disagree	5 (1.1%)
Disagree	3 (0.6%)
Neutral	26 (5.5%)
Agree	97 (20.6%)
Strongly agree	339 (72.1%)
It is necessary to visit a doctor when noticing a rapid increase in skin redness, accompanied by elevated temperature, chills, swelling, and pain in the skin	
Strongly disagree	3 (0.6%)
Disagree	9 (1.9%)
Neutral	28 (6.0%)
Agree	79 (16.8%)
Strongly agree	351 (74.7%)
More education and awareness of cellulitis should be provided	
Strongly disagree	4 (0.9%)
Disagree	2 (0.4%)
Neutral	26 (5.5%)
Agree	91 (19.4%)
Strongly agree	347 (73.8%)

## Discussion

The current study aimed to assess the public awareness of cellulitis in the Al-Qunfudhah region, Saudi Arabia. In cases where patients lack knowledge of cellulitis, they are unlikely to alter risk factors to avoid recurrent bouts [21]. A single, brief qualitative study with secondary care patients receiving cellulitis treatment has been conducted. Participants expressed not getting enough assistance or information, as well as not always knowing what to do to stop recurrences [22]. This can be the result of conflicting advice on cellulitis prevention. The current study also aimed to investigate the prevalence and characteristics of cellular tissue inflammation in the Al-Qunfudhah region.

With regard to the prevalence of cellulitis, the current study revealed that about one-fifth of the participants had ever been diagnosed with cellulitis, mainly in their extremities (lower limbs and upper limbs), which is in line with the literature that revealed cellulitis usually affects the lower extremities more commonly compared with other parts of the body [23,24]. Old age, male gender, and employment were significantly associated with a higher frequency of cellulitis among study participants. Literature shows that cellulitis is observed most frequently among middle-aged and older adults, which is consistent with the current study findings [9,25]. Worldwide, about 200 cases of cellulitis per 100,000 patients are registered per year, which is much lower than assessed in the current study [6]. A much lower prevalence was reported by Hailu et al. [26], where an incidence rate of 1.8% was assessed, but similar to the current study, most patients were males. Also, in the USA, 1.4% of cellulitis cases are noted in hospital admissions [27]. In Saudi Arabia, the incidence of skin and soft-tissue infections was 1.67% [28]. Another study revealed that cellulitis accounted for 23.5% of general surgical admission during Hajj Pilgrims [29]. The estimated high prevalence in the current study may be due to subjective methods used for identifying the disease frequency based on participants' judgments, who may overreport any skin condition or dermatitis as cellulitis.

With regard to public awareness of cellulitis, the current study revealed that about half of the study participants heard about cellulitis, with most participants correctly defining the condition. Also, about half of them identified the clinical presentation, diagnostic techniques, management methods, and associated complications of neglected cases. Pain, local redness, and warmth were identified by more than three-fourths of the study participants. Also, bloodstream infections were the most commonly associated complication with tissue death. The Internet, healthcare providers, and TV were the main sources of information about cellulitis. Old age, high education, and employment were the significant factors associated with high awareness levels. In contrast to the current study, Teasdale et al. [21] reported low awareness of cellulitis among their study participants. Also, the study revealed that participants were astonished that they had never heard of cellulitis and that they had not received advice or leaflets giving self-care information. Some sought information from the Internet and found this confusing. Another study by Qadir et al. [30] among students revealed that 93.5% of students knew that cellulitis was a bacterial disease, 83.1% knew that it could be treated medically, and 5.1% reported surgical treatment. In Saudi Arabia, no previous studies assessed public awareness of cellulitis, but Alshaalan et al. [31] assessed public awareness of skin diseases, and none of them reported cellulitis as one of the skin-related diseases. Regarding public attitudes towards cellulitis, it was found that most of the study participants agreed with the role of healthcare staff in providing more information to raise public awareness of the disease, which will help in disease prevention with a lower burden. This manuscript offers some strength points as it is the first work to discuss awareness of cellulitis health problems in a specific area (Al-Qunfudhah Governorate), raising their knowledge, especially in high-risk patients. Meanwhile, it is important to explore the leading role of healthcare provider teams in upgrading the culture of the Al-Qunfudhah population regarding the care of cellulitis cases.

Study limitations

This study encountered several limitations. Firstly, it used a small sample size of participants due to the limited population of Al-Qunfudhah Governorate, which may limit its generalization. Secondly, it is self-reported, which may be affected by sample bias because it depends on social media, which may be influenced by the availability of the Internet and social media accounts. Thirdly, the well-structured questionnaire limits the depth of participants' understanding of knowledge and attitudes towards cellulitis. Lastly, this study cannot differentiate between variables' causal relationships, which limits the investigation of the association between cause and effect.

## Conclusions

The current study revealed that the estimated prevalence of cellulitis was higher than reported in the literature due to the subjective assessment used in the current study. Additionally, the study showed high awareness among participants regarding all aspects of cellulitis, including the nature of the disease, clinical presentation, complications, diagnosis, and management. Higher education and employment were associated with higher awareness levels. Study participants emphasized the role of healthcare staff in improving public awareness and perception of cellulitis to minimize the disease-associated burden. Periodic health education campaigns are recommended to improve public healthcare awareness of skin-related disorders and preventable diseases such as cellulitis. More effort should be made by healthcare staff, as they are a trusted source of information for the community.
